# A comprehensive study on bilingual and multilingual speech emotion recognition using a two-pass classification scheme

**DOI:** 10.1371/journal.pone.0220386

**Published:** 2019-08-15

**Authors:** Panikos Heracleous, Akio Yoneyama

**Affiliations:** Education and Medical ICT Laboratory, KDDI Research, Inc., 2-1-15 Ohara, Fujimino-shi, Saitama 356-8502 Japan; Manukau Institute of Technology, NEW ZEALAND

## Abstract

Emotion recognition plays an important role in human-computer interaction. Previously and currently, many studies focused on speech emotion recognition using several classifiers and feature extraction methods. The majority of such studies, however, address the problem of speech emotion recognition considering emotions solely from the perspective of a single language. In contrast, the current study extends monolingual speech emotion recognition to also cover the case of emotions expressed in several languages that are simultaneously recognized by a complete system. To address this issue, a method, which provides an effective and powerful solution to bilingual speech emotion recognition, is proposed and evaluated. The proposed method is based on a two-pass classification scheme consisting of spoken language identification and speech emotion recognition. In the first pass, the language spoken is identified; in the second pass, emotion recognition is conducted using the emotion models of the language identified. Based on deep learning and the i-vector paradigm, bilingual emotion recognition experiments have been conducted using the state-of-the-art English IEMOCAP (four emotions) and German FAU Aibo (five emotions) corpora. Two classifiers along with i-vector features were used and compared, namely, fully connected deep neural networks (DNN) and convolutional neural networks (CNN). In the case of DNN, 64.0% and 61.14% unweighted average recalls (UARs) were obtained using the IEMOCAP and FAU Aibo corpora, respectively. When using CNN, 62.0% and 59.8% UARs were achieved in the case of the IEMOCAP and FAU Aibo corpora, respectively. These results are very promising, and superior to those obtained in similar studies on multilingual or even monolingual speech emotion recognition. Furthermore, an additional baseline approach for bilingual speech emotion recognition was implemented and evaluated. In the baseline approach, six common emotions were considered, and bilingual emotion models were created, trained on data from the two languages. In this case, 51.2% and 51.5% UARs for six emotions were obtained using DNN and CNN, respectively. The results using the baseline method were reasonable and promising, showing the effectiveness of using i-vectors and deep learning in bilingual speech emotion recognition. On the other hand, the proposed two-pass method based on language identification showed significantly superior performance. Furthermore, the current study was extended to also deal with multilingual speech emotion recognition using corpora collected under similar conditions. Specifically, the English IEMOCAP, the German Emo-DB, and a Japanese corpus were used to recognize four emotions based on the proposed two-pass method. The results obtained were very promising, and the differences in UAR were not statistically significant compared to the monolingual classifiers.

## Introduction

Automatic recognition of human emotions is of vital importance in human-computer interaction and its applications [1]. Applications include human-robot communication, when robots respond to humans according to the detected emotions, implementation in call centers to detect the caller’s emotional state in cases of emergency, identifying the level of a customer’s satisfaction, medical analysis, and education. Emotion recognition can be conducted using facial expressions, verbal communication, text, electroencephalography (EEG) signals, or a combination of multiple modalities. Furthermore, emotion recognition can identify emotions solely in relation to a single language, or can simultaneously recognize emotions expressed through several languages. Although many studies on monolingual emotion recognition have been published, multilingual emotion recognition is still an open research area. Therefore, in the current study, comprehensive experiments and analysis of bilingual and multilingual emotion recognition based on speech, using English, German, and Japanese corpora are reported. For classification, deep neural networks fed with i-vector [2] features are used.

Previous studies on speech emotion recognition reported methods based on Gaussian mixture models (GMMs) [3, 4], hidden Markov models (HMMs) [5], and support vector machines (SVM) [6–8]. Other studies demonstrate speech emotion recognition based on neural networks [9, 10] and deep neural networks (DNN) [11, 12]. Furthermore, in [13], audio-visual emotion recognition has also been presented.

The majority of studies in speech emotion recognition focused solely on a single language, while cross-corpus or multilingual speech emotion recognition has been addressed in only a few studies. In [14], experiments on emotion recognition are described using speech corpora collected from American English and German interactive voice response systems, and the optimal set of features for mono-, cross-, and multilingual anger recognition were computed. Cross-language speech emotion recognition based on HMMs and GMMs is reported in [15]. Four speech databases for cross-corpus classification, with realistic emotions and a large acoustic feature vector are reported in [16]. Similarly, cross-lingual speech emotion recognition is introduced in [17–19].

The current study approaches the problem of bilingual and multilingual speech emotion recognition by exploiting spoken language identification. A method that integrates spoken language identification and speech emotion recognition into a complete system is proposed. A two-pass classification scheme is demonstrated to allow the selection of appropriate emotion models according to the language identified in the first pass. State-of-the-art classifiers are used in both passes namely, DNN and convolutional neural networks (CNN) [20, 21]. Considering the success of i-vectors in many speech applications, in the proposed method, i-vectors are used as input features. The well-known and effective mel-frequency cepstral coefficients (MFCC) [22] concatenated with shifted delta cepstral (SDC) coefficients [23, 24] are used to extract the i-vectors used in the experiments. SDC coefficients were originally applied in spoken language identification due to superior performance compared to the sole use of MFCC features. In the current study, in addition to spoken language identification, SDC coefficients are also used for speech emotion recognition. In the current study, comprehensive investigation and analysis on bilingual and multilingual speech emotion recognition are conducted. Additionally, another method based on deep learning, which uses common bilingual emotion models and without spoken language identification, is introduced and compared with the proposed method. The improvements when using SDC coefficients are also described and the differences when using MFCC features only are shown.

Multilingual speech emotion recognition based on spoken language identification was also reported in [25]. In that specific study, i-vectors and a Gaussian linear classifier were applied for spoken language identification. For emotion recognition, low-level descriptors (LLD) and SVM were used. The results showed improvements in most cases using spoken language identification (nine out of twelve conditions). In contrast, the current study is based on advanced classifiers such as DNN and CNN integrated with i-vectors for both language identification and speech emotion recognition. Although, i-vectors have previously been used in speech emotion recognition, to date, the integration of deep learning (DL) and i-vectors in the case of very limited training data has not been investigated exhaustively. Also, the case of limited training i-vectors and DL in spoken language identification has been examined in only a few studies [26, 27]. Furthermore, in the current study, the FAU Aibo [28] and the IEMOCAP [29] state-of-the-art emotional corpora are used for bilingual emotion recognition based on DNN and i-vectors. In addition to the DNN and i-vector-based method, another method is also reported which uses CNN in conjunction with i-vectors.

The current study was further extended to address recognition of emotions in three languages. Specifically, experiments were conducted on multilingual emotion recognition using the English IEMOCAP, the German Emo-DB [30], and a Japanese emotional corpus [31]. The three speech corpora were collected under similar conditions and therefore, the experiments are more realistic as they also eliminate possible mismatches between the English IEMOCAP (i.e. adult’s speech) and FAU-Aibo (i.e. children’s speech).

Automatic language identification is a process whereby a spoken language is identified automatically. Applications of language identification include, but are not limited to, speech-to-speech translation systems, re-routing incoming calls to native speaker operators at call centers, and speaker diarization. Because of the importance of spoken language identification in real applications, many studies have addressed this issue. The approaches reported are categorized into the acoustic-phonetic approach, the phonotactic approach, the prosodic approach, and the lexical approach [32]. In phonotactic systems [32, 33], sequences of recognized phonemes obtained from phone recognizers are modeled. In [34], a typical phonotactic language identification system is used, where a language dependent phone recognizer is followed by parallel language models (PRLM). In [35], a universal acoustic characterization approach to spoken language recognition is proposed. Another method based on vector-space modeling is reported in [32, 36], and presented in [37].

In acoustic modeling-based systems, different features are used to model each language. Earlier language identification studies reported methods based on neural networks [38, 39]. Later, the first attempt at using deep learning was also reported [40]. Deep neural networks for language identification were used in [41]. The method was compared with i-vector-based classification, linear logistic regression, linear discriminant analysis-based (LDA), and Gaussian modeling-based classifiers. In the case of a large amount of training data, the method demonstrated its superior performance. When limited training data were used, the i-vector yields the best identification rate. In [42] a comparative study on spoken language identification using deep neural networks was presented by the authors. Other methods based on DNN and recurrent neural networks (RNN) were presented in [43, 44]. In [45], the authors reported experiments on language identification using i-vectors and conditional random fields (CRF) [46–49]. The i-vector paradigm for language identification with SVM [50] was also applied in [51]. SVM with local Fisher discriminant analysis was used in [52]. Although significant improvements in LID have been achieved using phonotactic approaches, most state-of-the-art systems still rely on acoustic modeling.

## Materials and methods

### Evaluation metrics

In the current study, recall, precision, F1-score and unweighted average recall (UAR) are used as evaluation metrics. Based on Table 1, the metrics in binary classification case are computed as follows:

$$\begin{array}{c} \text{Recall}=\frac{TP}{TP+FN} \end{array}$$ (1)

$$\begin{array}{c} \text{Precision}=\frac{TP}{TP+FP} \end{array}$$

$$\begin{array}{c} F1-\text{score}=2\times \frac{Precision\times Recall}{Precision+Recall} \end{array}$$

$$\begin{array}{c} UAR=\frac{1}{N}\sum_{i=1}^{N}Recall_{i}\;\;\;\;\text{N}\;\text{number}\;\text{of}\;\text{classes} \end{array}$$

**Table 1:** Recall, precision, and F1-score in the binary case.

	Predicted Class		
		(+)	(-)
Actual Class	(+)	True Positives (TP)	False Negatives (FN)
	(-)	False Positives (FP)	True Negatives (TN)

The metrics shown in Eq 1 can be generalized for multi-class classification by considering the individual classes, accordingly.

### Data

For bilingual emotion recognition, the English Interactive Emotional Dyadic Motion Capture (IEMOCAP) and the spontaneous German FAU Aibo emotional databases are used. The IEMOCAP database is an acted, multimodal and multispeaker database, collected at the SAIL lab of the University of Southern California. It contains 12 hours of audiovisual data produced by ten actors. Specifically, the IEMOCAP database includes video, speech, motion capture of facial expressions, and text transcriptions. The IEMOCAP database is annotated by multiple annotators into several categorical labels, such as anger, happiness, sadness, and neutrality, as well as dimensional labels such as valence, activation and dominance. In the current study, categorical labels were used to classify the emotional states of *neutral*, *happy*, *angry*, and *sad*. To avoid unbalanced data, 250 training utterances and for testing 50 utterances randomly selected for each emotion were used.

The FAU Aibo corpus consists of 9 hours of German speech derived from 51 children aged 10-13 years interacting with Sony’s pet robot Aibo. The spontaneous emotional children’s speech has been recorded using a close-talking microphone. The data are annotated with 11 emotion categories by five human labelers on the word level. In the current study, the FAU Aibo data are used for classification of the *angry*, *emphatic*, *joyful*, *neutral*, and *rest* emotional states. To use balanced training and test data, 590 training utterances and 299 test utterances randomly selected for each emotion were used.

The German database used was the Berlin Emo-DB database, which includes seven emotional states: anger, boredom, disgust, anxiety, happiness, sadness, and neutral speech. The utterances were produced by ten professional German actors (five female and five male) uttering ten sentences with an emotionally neutral content but expressed with the seven different emotions. The actors produced 69 frightened, 46 disgusted, 71 happy, 81 bored, 79 neutral, 62 sad, and 127 angry emotional sentences. In the multilingual experiment on three languages, the emotions happy, neutral, sad, and angry were considered. For each emotion, 40 instances were used for training, and 22 instances were used for testing.

Four professional female actors simulated Japanese emotional speech. These comprised neutral, happy, angry, and sad emotional states. Fifty-one utterances for each emotion were produced by each speaker. The sentences were selected from a Japanese book for children. The data were recorded at 48 kHz and down-sampled to 16 kHz, and they also contained short and longer utterances varying from 1.5 seconds to 9 seconds. Twenty-eight utterances from each speaker and emotion were used for training and 20 utterances from each speaker and emotion were used for testing. In total, 512 utterances were used for training, and 256 utterances were used for testing. The remaining utterances were excluded due to poor speech quality.

Table 2 shows the emotions used in bilingual emotion recognition using the IEMOCAP and FAU Aibo corpora when spoken language identification was not used (i.e., common bilingual emotion models). Six emotions were considered namely, *happy*, *angry*, *sad*, *neutral*, *emphatic*, and *rest*. For training, 450 utterances were used, and for testing, 100 utterances for each emotion were used. The training and testing data included randomly selected utterances from both the English and German corpora. In the case of spoken language identification in the first-pass, the same data as that used in speech emotion recognition were used. For each language, the utterances of all emotions were pooled to create the training and test data for the language identification task.

**Table 2:** Emotions considered in bilingual emotion recognition with a common model set.

Monolingual emotions		Bilingual emotion
IEMOCAP	FAU Aibo	
Happy	Joyful	Happy
Angry	Angry	Angry
Sad	-	Sad
Neutral	Neutral	Neutral
-	Emphatic	Emphatic
-	Rest	Rest

### Shifted delta cepstral (SDC) coefficients

Previous studies showed that language identification performance is improved by using SDC feature vectors, which are obtained by concatenating delta cepstra across multiple frames. The SDC features are described by the *N* number of cepstral coefficients, *d* time advance and delay, *k* number of blocks concatenated for the feature vector, and *P* time shift between consecutive blocks. For each SDC final feature vector, *kN* parameters are used. In contrast, in the case of conventional cepstra and delta cepstra feature vectors, *2N* parameters are used. The SDC is calculated as follows:

$$\begin{array}{c} \Delta c(t+iP)=c(t+iP+d)-c(t+iP-d) \end{array}$$ (2)

**Fig 1:**
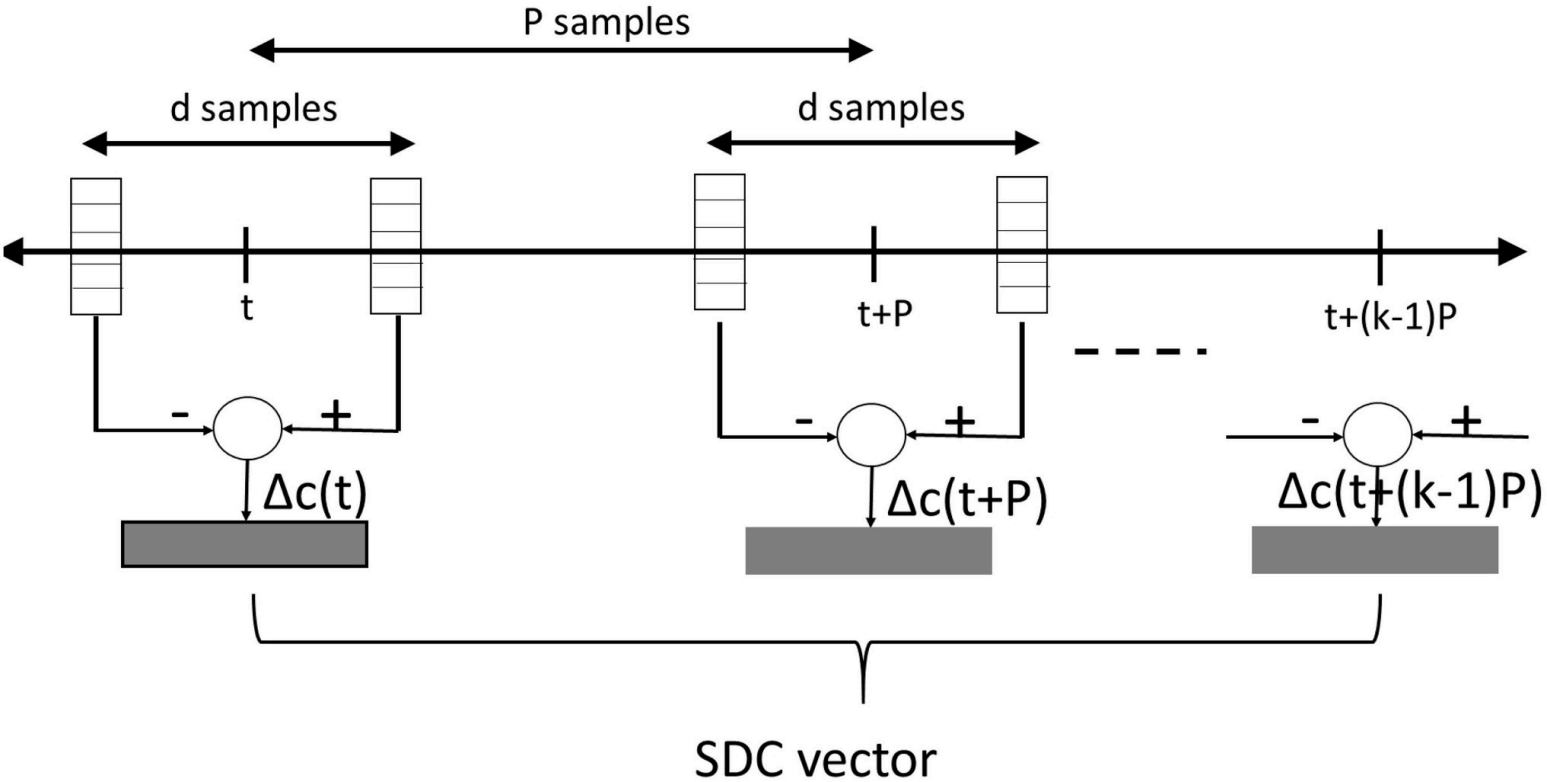
Computation of shifted delta cepstral (SDC) coefficients.

The final vector at time *t* is given by the concatenation of all Δ*c*(*t* + *iP*) for all 0 ≤ *i* < *k*, where *c*(*t*) is the original feature value at time *t*. In the current study, SDC coefficients were used not only in spoken language identification, but also in emotion classification. Fig 1 shows the computation procedure of the SDC coefficients.

### Feature extraction

In automatic speech recognition, speaker recognition, and language identification MFCC features are among the most popular and widely used acoustic features. Therefore, in modeling the languages being identified, this study also used 12 MFCC features, concatenated with SDC coefficients to form feature vectors of length 112. The MFCC features were extracted every 10 ms using a window-length of 20 ms. The extracted acoustic features were used to construct the i-vectors used in emotion and spoken language identification modeling and classification.

### The i-vector paradigm

A widely used approach for speaker recognition is based on Gaussian mixture models (GMM) with universal background models (UBM). The individual speaker models are created using maximum a posteriori (MAP) adaptation of the UBM. In many studies, GMM supervectors are used as features. The GMM supervectors are extracted by concatenating the means of the adapted model.

The problem of using GMM supervectors is their high dimensionality. To address this issue, the i-vector paradigm was introduced which overcomes the limitations of high dimensionality. In the case of i-vectors, the variability contained in the GMM supervectors is modeled with a small number of factors, and the whole utterance is represented by a low dimensional i-vector of 100-400 dimension.

Considering language identification, an input utterance can be modeled as:

$$\begin{array}{c} M=m+\text{Tw} \end{array}$$ (3)

where **M** is the language-dependent supervector, **m** is the language-independent supervector, **T** is the total variability matrix, and **w** is the i-vector. Both the total variability matrix and language-independent supervector are estimated from the complete set of the training data. The same procedure is used to extract i-vectors used in speech emotion recognition.

### Classification approaches

#### Deep neural networks (DNN)

DNN is an important method for machine learning, and has been applied in many areas. A DNN is a feed-forward neural network with many (i.e., more than one) hidden layers. The main advantage of DNNs compared to shallow networks is the better feature expression and the ability to perform complex mapping. Deep learning is behind several of the most recent breakthroughs in computer vision, speech recognition, and agents that achieved human-level performance in games such as go and poker. In the current study, four hidden layers with 64 units and *ReLu* activation function are used. On top, a fully-connected *Softmax* layer is added. The number of batches is set to 512, and 500 epochs are used.

#### Convolutional neural networks (CNN)

A convolutional neural network is a special variant of the conventional deep neural network, and consists of alternating convolution and pooling layers. Convolutional neural networks have been successfully applied to sentence classification [53], image classification [54], facial expression recognition [55], and in speech emotion recognition [56]. In [57] bottleneck features for language identification are extracted using CNNs.

**Fig 2:**
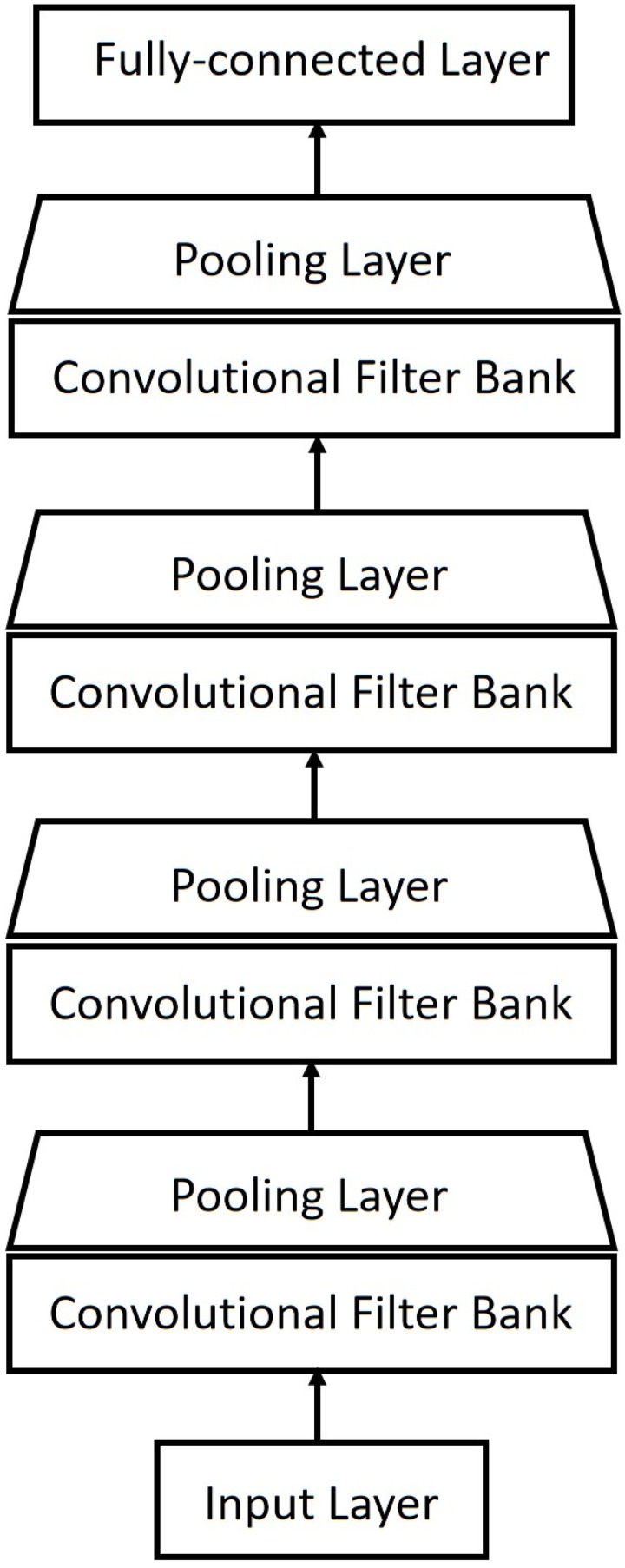
Architecture of the proposed convolutional neural networks-based classifier.

In the proposed CNN architecture, four convolutional layers with 64 5 × 5 filters and the *ReLu* activation function were used. Each convolutional layer is followed by a max-pooling layer with width = 2 × 2. On top, a fully connected *Softmax* layer was used. The batch size was set to 64, and the dropout probability was set to 0.25. The epochs number was 200. Fig 2 shows the architecture of the proposed method.

## Results

### Spoken language identification using emotional data

In the first pass of the proposed method for emotion recognition, a spoken language identification module is implemented. The task of this module is to identify the spoken language and to switch to the appropriate emotion models. For classification, DNN and CNN trained with IEMOCAP and FAU Aibo databases are used. The system is fed with i-vectors constructed from concatenated MFCC and SDC features. Although the proposed method focuses on only two languages, the system can deal with additional languages of interest. The performance of the first pass significantly affects the overall classification accuracy of the emotions included in the IEMOCAP and FAU Aibo databases. Therefore, it is of vital importance to apply powerful classification approaches and effective feature extraction methods. To address this issue, in the current study state-of-the-art DNN and CNN, in conjunction with i-vectors features are used.

Table 3 shows the identification rates when using DNN and CNN, respectively. As shown, when using supplemented with SDC coefficients the identification rate are is 100.0% in all cases. Without SDC coefficients, the rates in some cases are slightly lower. Results also show that the same identification rates are obtained when using DNN and CNN, respectively.

**Table 3:** Spoken language identification rates [%] using English and German emotional speech data.

Features used in i-vectors extraction	Classification Method			
	DNN		CNN	
	English	German	English	German
MFCC	100.0	99.0	100.0	99.0
MFCC+SDC	100.0	100.0	100.0	100.0

The results show the effectiveness of using deep learning and i-vectors for spoken language identification. Note, however, that only two languages are identified and very high rates may be expected. Another possible reason for the high identification rates obtained may be the mismatch between the two corpora (adult’s speech vs children’s speech). Also the recording environment and conditions may differ resulting in higher classification rates. The problems of speaker, environment, acoustic, and technology based mismatch in speech, speaker, and language recognition have been addressed and discussed in details in [58]. In that study, the authors suggested some solutions to enable the collection of more realistic data. On the other hand, language identification using emotional data was not associated with additional difficulty compared to normal speech. In general, language identification is conducted using normal speech. In the proposed method, however, emotional speech is used to identify the language in the first pass. The results obtained show that even in emotional speech, information about the language spoken is included in a way similar to normal speech.

### Bilingual emotion recognition based on two-pass classification scheme

This section presents the results of bilingual speech emotion recognition using the proposed two-pass classification scheme. The results also show the differences when DNN and CNN were used. Furthermore, the improvements when SDC coefficients are used in conjunction with MFCC features are demonstrated.

#### Results using the English IEMOCAP corpus

Tables 4 and 5 show the recalls for the English IEMOCAP data when using DNN and CNN, respectively. As shown, *angry* and *sad* emotions have the highest recalls in both DNN and CNN followed by the emotions *neutral* and *happy*. The order of individual recalls is consistent with the order reported in [59]. The UARs in the case of using MFCC features only were 56.5% and 55.5% for DNN and CNN, respectively. When SDC coefficients were also used, the UARs for DNN and CNN were 64.0% and 62.0%, respectively. Note that when MFCC features were used in conjunction with SDC coefficients, the UAR for *neutral* emotion in DNN, and for *happy* emotion in CNN decreased. However, the UARs when SDC coefficients were concatenated with MFCC features show relative improvements of 17.2% and 13.7% based on DNN and CNN classifiers, respectively. These results are very promising and demonstrate the effectiveness of the proposed method for bilingual speech emotion recognition. The results obtained are even superior or very similar to those obtained in studies using the IEMOCAP corpus for monolingual speech emotion recognition [60–62].

**Table 4:** Recalls for speech emotion recognition using IEMOCAP and DNN.

Features used in i-vectors extraction	Emotions				
	Neutral	Happy	Angry	Sad	UAR
MFCC	52.0	42.0	70.0	62.0	56.5
MFCC+SDC	48.0	44.0	88.0	76.0	64.0

**Table 5:** Recalls for speech emotion recognition using IEMOCAP and CNN.

Features used in i-vectors extraction	Emotions				
	Neutral	Happy	Angry	Sad	UAR
MFCC	46.0	40.0	66.0	70.0	55.5
MFCC+SDC	48.0	36.0	88.0	76.0	62.0

Tables 6 and 7 show the precisions obtained when using DNN and CNN. The precisions are also compared when using MFCC features and MFCC features with SDC coefficients, respectively. The results show higher precision when SDC coefficients were used, and also the superior performance of DNN.

**Table 6:** Precision of speech emotion recognition using IEMOCAP and DNN.

Features used in i-vectors extraction	Emotions				
	Neutral	Happy	Angry	Sad	Average
MFCC	45.61	51.22	66.04	63.27	56.54
MFCC+SDC	51.06	57.89	77.19	65.52	62.92

**Table 7:** Precision of speech emotion recognition using IEMOCAP and CNN.

Features used in i-vectors extraction	Emotions				
	Neutral	Happy	Angry	Sad	Average
MFCC	45.10	47.62	68.75	59.32	55.20
MFCC+SDC	48.98	54.55	73.33	65.52	60.60

Tables 8 and 9 show F1-scores obtained when using DNN and CNN. As shown, higher F1-scores were obtained using DNN compared with CNN. The results also show improved scores when SDC coefficients were concatenated with MFCC features.

**Table 8:** F1-scores for speech emotion recognition using IEMOCAP and DNN.

Features used in i-vectors extraction	Emotions				
	Neutral	Happy	Angry	Sad	Average
MFCC	48.60	46.15	67.96	62.63	56.34
MFCC+SDC	49.48	50.00	82.24	70.37	63.02

**Table 9:** F1-scores for speech emotion recognition using IEMOCAP and CNN.

Features used in i-vectors extraction	Emotions				
	Neutral	Happy	Angry	Sad	Average
MFCC	45.54	43.48	67.35	64.22	55.15
MFCC+SDC	48.48	43.37	80.00	70.37	60.56

Tables 10 and 11 show the confusion matrices when using DNN and CNN, respectively. As shown, in both cases, similar tendencies are observed. The emotions *neutral* and *happy* show a high number of confusions. The emotions *angry* and *sad* show the lowest number of misclassifications.

**Table 10:** Confusion matrix [%] using IEMOCAP and DNN with MFCC/SDC features.

	Neutral	Happy	Angry	Sad
Neutral	**48.0**	20.0	12.0	20.0
Happy	30.0	**44.0**	10.0	16.0
Angry	4.0	4.0	**88.0**	4.0
Sad	12.0	8.0	4.0	**76.0**

**Table 11:** Confusion matrix [%] using IEMOCAP and CNN with MFCC/SDC features.

	Neutral	Happy	Angry	Sad
Neutral	**48.0**	18.0	14.0	20.0
Happy	34.0	**36.0**	14.0	16.0
Angry	4.0	4.0	**88.0**	4.0
Sad	12.0	8.0	4.0	**76.0**

#### Results using the German FAU AIBO corpus

Tables 12 and 13 show the recalls for bilingual speech emotion recognition when using the German FAU Aibo corpus. In this case, five emotions are classified. The results show the comparisons when using MFCC features and MFCC features concatenated with SDC coefficients. Furthermore, DNN and CNN classifiers are compared. The results show that when SDC coefficients are used, the recalls are significantly higher compared with the recalls when MFCC features are used on their own. Specifically, in the case of DNN, the UAR improves from 38.99% to 61.14%, and in the case of using CNN, the UAR improves from 39.53% to 59.80%. In contrast to the English IEMOCAP corpus, when the German FAU Aibo corpus was used, all emotions show higher recalls when SDC coefficients are concatenated with MFCC features. The emotion *joyful* has the highest recall, while the emotion *rest* has the lowest recall. The results obtained are superior or comparable to those reported in similar studies [63–65].

**Table 12:** Recalls for speech emotion recognition using FAU Aibo and DNN.

Features used in i-vectors extraction	Emotions					
	Angry	Emphatic	Joyful	Neutral	Rest	UAR
MFCC	40.47	42.47	48.16	28.76	35.12	38.99
MFCC+SDC	63.55	63.88	68.90	60.20	49.16	61.14

**Table 13:** Recalls for speech emotion recognition using FAU Aibo and CNN.

Features used in i-vectors extraction	Emotions					
	Angry	Emphatic	Joyful	Neutral	Rest	UAR
MFCC	46.49	35.12	53.51	33.78	28.7	39.53
MFCC+SDC	55.52	62.88	71.24	68.23	41.14	59.80

Tables 14 and 15 show the precisions when using DNN and CNN in the case of the German FAU Aibo corpus. It can be clearly seen that when using SDC coefficients concatenated with MFCC features, the precisions increased. It can also be seen that DNN has superior performance in the case of FAU Aibo, too.

**Table 14:** Precision of speech emotion recognition using FAU Aibo and DNN.

Features used in i-vectors extraction	Emotions					
	Angry	Emphatic	Joyful	Neutral	Rest	Average
MFCC	41.02	33.60	55.38	37.55	31.53	39.82
MFCC+SDC	64.85	61.41	69.36	62.50	48.04	61.23

**Table 15:** Precision of speech emotion recognition using FAU Aibo and CNN.

Features used in i-vectors extraction	Emotions					
	Angry	Emphatic	Joyful	Neutral	Rest	Average
MFCC	41.12	35.35	52.81	35.07	31.97	39.26
MFCC+SDC	67.76	58.93	69.38	58.79	44.40	59.85

Tables 16 and 17 show the F1-scores when using DNN and CNN in the case of the German FAU Aibo corpus. The results show the same tendency as in recall and precision.

**Table 16:** F1-scores for speech emotion recognition using FAU Aibo and DNN.

Features used in i-vectors extraction	Emotions					
	Angry	Emphatic	Joyful	Neutral	Rest	Average
MFCC	40.74	37.52	51.52	32.58	33.23	39.12
MFCC+SDC	64.19	62.62	69.13	61.33	48.60	61.17

**Table 17:** F1-scores for speech emotion recognition using FAU Aibo and CNN.

Features used in i-vectors extraction	Emotions					
	Angry	Emphatic	Joyful	Neutral	Rest	Average
MFCC	43.64	35.23	53.16	34.41	30.28	39.34
MFCC+SDC	61.03	60.84	70.34	63.16	42.71	59.61

Tables 18 and 19 show the confusion matrices for bilingual emotion recognition using the German FAU Aibo data. The results obtained for bilingual speech recognition using the English corpus and the German corpus clearly indicate the effectiveness of the proposed two-pass classification approach. The UAR using DNN was 64.0% and 61.14% for the English and German corpora, respectively. When using CNN, the average classification rates obtained for English and German were 62.0% and 59.8%, respectively.

**Table 18:** Confusion matrix [%] using FAU Aibo and DNN with MFCC/SDC features.

	Angry	Emphatic	Joyful	Neutral	Rest
Angry	**63.55**	14.38	6.69	5.02	10.37
Emphatic	15.05	**63.88**	0.33	14.38	6.35
Joyful	3.68	2.34	**68.9**	4.35	20.74
Neutral	3.34	14.38	6.35	**60.2**	15.72
Rest	12.37	9.03	17.06	12.37	**49.16**

**Table 19:** Confusion matrix [%] using FAU Aibo and CNN with MFCC/SDC features.

	Angry	Emphatic	Joyful	Neutral	Rest
Angry	**55.52**	18.06	7.02	6.35	13.04
Emphatic	11.37	**62.88**	0.33	18.39	7.02
Joyful	2.68	2.68	**71.24**	5.69	17.73
Neutral	1.0	13.04	4.01	**68.23**	13.71
Rest	11.37	10.03	20.07	17.39	**41.14**

### Bilingual emotion recognition using a common model set

This section presents the results for bilingual emotion recognition using a common emotion model set. In this experiment, data from both the IEMOCAP and FAU Aibo corpora are used in conjunction to train the emotion models. Three emotion models are trained using both data (*happy, angry, neutral*), two emotion models are trained using the FAU Aibo data (*emphatic, rest*), and another emotion model is trained using the IEMOCAP data (*sad*).

Table 20 shows the recalls using a common emotion model set and DNN. As shown, the emotions *emphatic, rest, sad* have the highest recalls. This is attributable to the fact that for these emotion models, monolingual data were used. The UAR using only MFCC features was 48.33%, and when SDC coefficients were also used, the UAR increased to 51.17%.

**Table 20:** Recalls for speech emotion recognition using a common model set and DNN.

Features used in i-vectors extraction	Emotions					
	Angry	Emphatic	Happy	Neutral	Rest	Sad
MFCC	37.0	61.0	33.0	26.0	37.0	96.0
MFCC+SDC	51.0	54.0	26.0	29.0	51.0	96.0

Table 21 shows the recalls when CNN was used. As shown, the same tendency is observed when using DNN. The UAR using MFCC features was 47.83%, and when SDC coefficients were also used the UAR increased to 51.50%.

**Table 21:** Recalls for speech emotion recognition using a common model set and CNN.

Features used in i-vectors extraction	Emotions					
	Angry	Emphatic	Happy	Neutral	Rest	Sad
MFCC	39.0	60.0	42.0	25.0	29.0	92.0
MFCC+SDC	54.0	62.0	24.0	37.0	40.0	92.0

The recalls using a common model set are lower compared with two-pass bilingual emotion recognition. Note, however, that in the case of using a common model set, six emotions were classified. Furthermore, the results achieved using DNN and CNN are very similar.

Tables 22 and 23 show the precisions when using DNN and CNN, respectively. In the case of DNN, the average precision using MFCC features only was 47.45%, and when SDC coefficients were also used a 50.43% precision was obtained. In the case of CNN, precisions of 46.58% and 49.98% were achieved using MFCC features and MFCC with SDC coefficients, respectively. As shown, the precisions for DNN and CNN were highly comparable, with DNN showing slightly better performance.

**Table 22:** Precision of speech emotion recognition using a common model set and DNN.

Features used in i-vectors extraction	Emotions					
	Angry	Emphatic	Happy	Neutral	Rest	Sad
MFCC	52.11	42.07	47.83	33.77	33.33	75.59
MFCC+SDC	53.13	59.34	36.62	44.62	34.46	74.42

**Table 23:** Precision of speech emotion recognition using a common model set and CNN.

Features used in i-vectors extraction	Emotions					
	Angry	Emphatic	Happy	Neutral	Rest	Sad
MFCC	52.7	43.17	44.21	30.86	31.87	76.67
MFCC+SDC	47.37	55.36	41.38	42.05	37.04	76.67

The F1-scores obtained when using a common emotion model set are shown in the Tables 24 and 25 when using DNN and CNN, respectively. When using DNN and MFCC features only, the average F1-score was 46.86%. When SDC coefficients were also used an average F1-score of 49.85% was obtained. In the case of using CNN, the average F1-score was 46.63% with MFCC features only, and 50.13% when SDC coefficients were concatenated. The results show, that for both DNN and CNN cases, comparable F1-scores were observed.

**Table 24:** F1-scores for speech emotion recognition using a common model set and DNN.

Features used in i-vectors extraction	Emotions					
	Angry	Emphatic	Happy	Neutral	Rest	Sad
MFCC	43.27	49.80	39.05	29.38	35.07	84.58
MFCC+SDC	52.04	56.54	30.41	35.15	41.13	83.84

**Table 25:** F1-scores for speech emotion recognition using a common model set and CNN.

Features used in i-vectors extraction	Emotions					
	Angry	Emphatic	Happy	Neutral	Rest	Sad
MFCC	44.83	50.21	43.08	27.62	30.37	83.64
MFCC+SDC	50.47	58.49	30.38	39.36	38.46	83.64

### Multilingual emotion recognition for English, German, and Japanese

In this section, the results for multilingual speech emotion recognition using corpora from three languages are presented. The experiments were based on the proposed two-pass classification scheme consisting of spoken language identification and speech emotion recognition. The method was evaluated in relation to the recognition of four emotions namely neutral, happy, angry, sad. In these experiments, unbalanced data were used from the IEMOCAP corpus. For the German and Japanese corpora, the training and test instances which were described previously in this paper were used. Table 26 shows the training and test instances for the English IEMOCAP.

**Table 26:** Training and test instances for the IEMOCAP corpus.

Instances	Emotions				
	Neutral	Happy	Angry	Sad	Total
Training	1139	397	735	723	2994
Test	569	198	368	361	1496
Total	1708	595	1103	1084	**4490**

Because of significant improvements achieved when SDC coefficients were used, in these experiments only MFCC concatenated with SDC coefficients were considered. Table 27 shows the confusion matrix of spoken language identification in the first-pass. As can be seen, the three languages were classified with high recalls. Specifically, the recall for the Japanese language was 96.48%, 97.43% for English, and 87.61% for German. The reason for the lower recall for German is the higher acoustic similarity between English and German. This was exacerbated by the high rate of confusion between English and German when German was the test language. The UAR obtained was 93.84%, which is a very promising result.

**Table 27:** Confusion matrix [%] of the spoken language identification in the first pass.

	Japanese	English	German
Japanese	96.48	3.52	0.0
English	0.41	97.43	2.16
German	0.88	11.51	87.61

**Fig 3:**
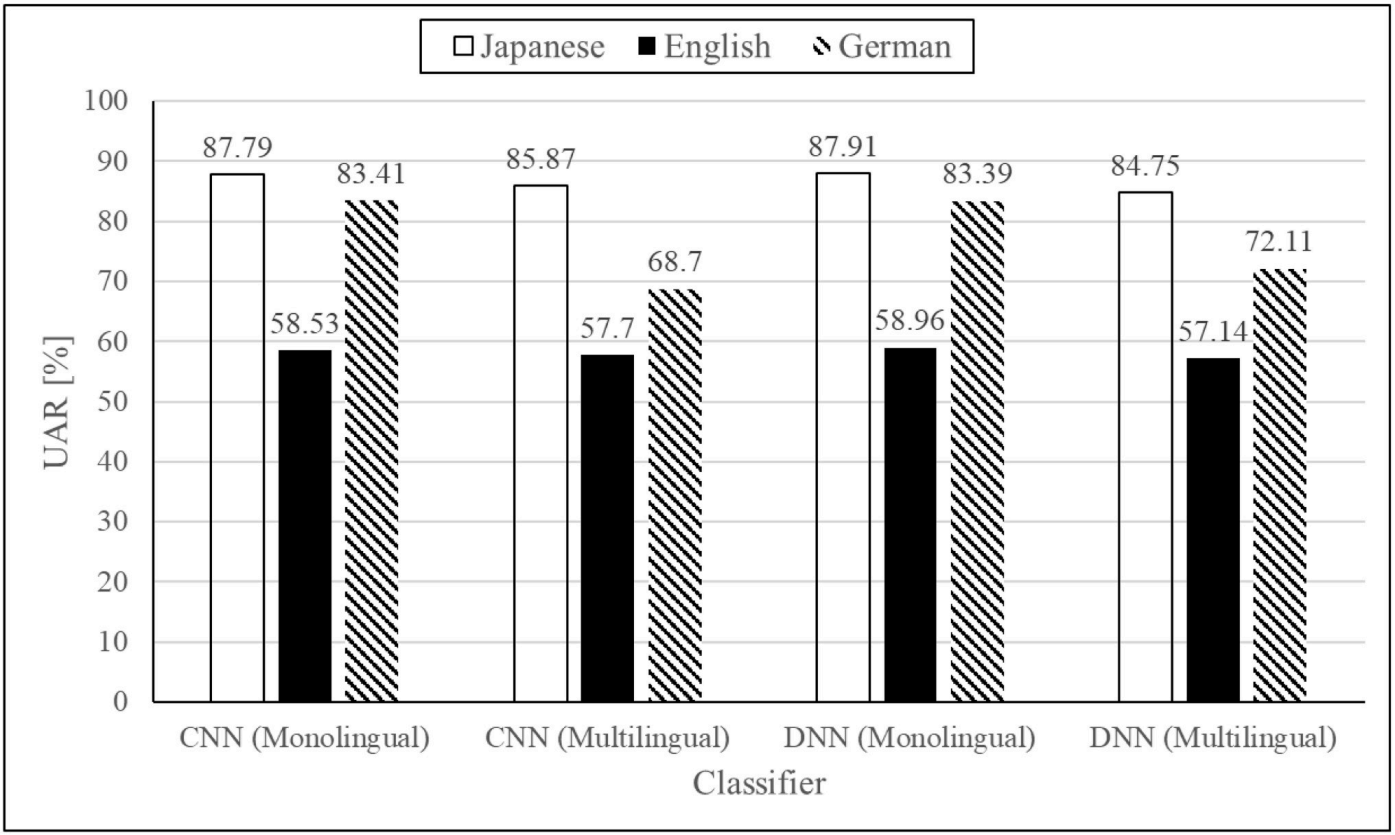
UARs for multilingual and monolingual emotion recognition for three languages.

Fig 3 shows the UARs achieved by monolingual classifiers along with the results achieved by the proposed two-pass multilingual approach. As can be seen, in the case of the English and Japanese corpora, the results obtained by monolingual and multilingual classifiers are highly comparable. In the case where the German corpus was used, the UAR for multilingual emotion recognition is lower because of the lower identification in the first pass. Compared to the recalls obtained in monolingual speech emotion recognition, the differences were considered not to be statistically significant. Performing the t-test, the two-tailed P value was 0.6116 in the case of CNN, and when using DNN the two-tailed P value was 0.6410.

## Discussion

The current study addresses the problem of multilingual speech emotion recognition. We conducted a comprehensive study that examined English and German emotional corpora for which the recognition of four and five emotions, respectively, were tested. Additionally, experiments on multilingual speech emotion recognition using three languages was also investigated. Although the current study considered only three languages, the same methodology and techniques can be extended to cover an arbitrary number of languages. In such studies, it is likely that performance will depend on the number of languages as well as on the acoustic similarities of the languages under consideration. Because the spoken language is identified in the first pass, acoustically similar languages will show a higher number of misclassifications resulting in decreased performance of the emotion recognition system. An interesting observation is the classification rate for the spoken language identified in the first pass using the emotional corpus. The results show perfect classification for IEMOCAP and FAU Aibo even where emotional data are used, and indicate that there are no additional difficulties compared to normal speech.

Regarding the features used in language identification and emotion recognition, several options (e.g., LLD, MFCC, i-vectors etc.) were considered when conducting the classification experiments. Given that i-vectors have been used successfully in several speech areas, and the small number of studies which integrate i-vectors and deep learning for language identification and emotion recognition where only very limited training data are available, it was decided that the current study would be based on the i-vector paradigm. To extract i-vectors, the well-known and very effective MFCC features were used. Furthermore, SDC coefficients were also applied in concatenation with MFCC features to investigate their effectiveness in both spoken language identification and emotion recognition. When SDC coefficients were also used, significant improvements in emotion classification rates were obtained.

In the experiments, the state-of-the-art English IEMOCAP and German FAU Aibo corpora were used for bilingual emotion recognition. Previously, several studies reported results using the two corpora, and many researchers continue to evaluate their methods using IEMOCAP or FAU Aibo data. Therefore, by using the two corpora, comparisons with similar studies are possible, though very often the experiments differ in terms of data selection and usage. In the current study, balanced data were used in both language identification and emotion recognition. In other studies, unbalanced training and test data were selected.

Another option that was considered was to use multilingual emotional speech corpora. Specifically, a multilingual emotional speech corpus for Slovenian, English, Spanish, and French language that was recorded under the IST project Interface “Multimodal Analysis/Synthesis System for Human Interaction to Virtual and Augmented environments” was also considered. However, that corpus faces the disadvantage of using data from two actors only producing a small amount of utterances. Another multilingual speech emotional corpus that was considered, was the EmoFilm corpus [66] consisting of 1115 utterances produced in English, Italian, and Spanish languages. This corpus, however, is not publicity available, and access to EmoFilm corpus was not possible. The proposed method was evaluated using DNN and CNN, and compared to a baseline method. Previously, only a few studies have reported spoken language identification and speech emotion recognition based on DNN and i-vectors. To our knowledge, however, the integration of CNN and i-vectors in these fields has not been investigated so far. In the current study, CNN was also integrated with i-vectors for language identification and emotion recognition. The main advantage of using CNN is that fewer parameters are required compared to DNN. As a result, CNN is more efficient in terms of memory and computational requirements. The results obtained using DNN and CNN showed comparable performance. Furthermore, even though only limited training data were used, the results obtained show that emotion recognition and language identification based on deep learning and i-vectors was still possible. These results confirm the previously reported results in [26, 27] for language identification using a small number of training i-vectors and deep learning. Therefore, the results obtained in the current study are of high importance and should prove to have great utility for society in general. Furthermore, the current study demonstrates that high classification rates can be obtained when deep neural networks and limited training i-vectors are used for speech emotion recognition.

## Conclusion

A method for bilingual and multilingual speech emotion recognition was presented. The proposed method is based on a two-pass classification scheme consisting of language identification and emotion recognition. In both passes, deep neural networks and i-vector features were used. The results obtained are very promising and superior or closely comparable to those obtained in similar studies on multilingual or monolingual speech emotion recognition using the same corpora. Currently, the proposed method is being extended to deal with a larger number of languages in order to investigate its effectiveness in multilingual speech emotion recognition. Furthermore, different feature extraction methods (e.g., combination of bottleneck features and i-vectors) are being considered.

## Supporting information

S1 File I-vector features for the Japanese emotional corpus. (ZIP)

## References

[1] Busso C, Bulut M, Narayanan SS. Toward Effective Automatic Recognition Systems of Emotion in Speech In: Gratch J, Marsella S, editors. Social emotions in nature and artifact: emotions in human and human-computer interaction. New York, NY, USA: Oxford University Press; 2013 p. 110–127.

[2] Dehak N, Kenny PJ, Dehak R, Dumouchel P, Ouellet P. Front-End Factor Analysis for Speaker Verification. IEEE Transactions on Audio, Speech, and Language Processing. 2011;19(4):788–798. 10.1109/TASL.2010.2064307

[3] Tang H, Chu SM, Johnson MH. Emotion Recognition From Speech Via Boosted Gaussian Mixture Models. in Proc of ICME. 2009; p. 294–297.

[4] Xu S, Liu Y, Liu X. Speaker Recognition and Speech Emotion Recognition Based on GMM. 3rd International Conference on Electric and Electronics (EEIC 2013). 2013; p. 434–436.

[5] Schuller B, Rigoll G, Lang M. Hidden Markov Model-based Speech Emotion Recognition. in Proc of the IEEE ICASSP. 2003;I:401–404.

[6] Pan Y, Shen P, Shen L. Speech Emotion Recognition Using Support Vector Machine. International Journal on Smart Home. 2012;6 (2):101–108.

[7] Hu H, Xu MX, Wu W. GMM Supervector Based SVM With Spectral Features for Speech Emotion Recognition. in Proc of ICASSP. 2007;IV:413–416.

[8] Chavhan Y, Dhore ML, Yesaware P. Speech Emotion Recognition Using Support Vector Machine. International Journal of Computer Applications (0975—8887). 2010;1, No. 20:6–9.

[9] Nicholson J, Takahashi K, Nakatsu R. Emotion Recognition in Speech Using Neural Networks. Neural Computing & Applications. 2000;9, Issue 4:290–296. 10.1007/s005210070006

[10] Shaw A, Vardhan RK, Saxena S. Emotion Recognition and Classification in Speech using Artificial Neural Networks. International Journal of Computer Applications (0975—8887). 2016;145, No.8:5–9. 10.5120/ijca2016910710

[11] Han K, Yu D, Tashev I. Speech Emotion Recognition Using Deep Neural Network and Extreme Learning Machine. in Proc of Interspeech. 2014; p. 223–227.

[12] Stuhlsatz A, Meyer C, Eyben F, Zielke T, Meier G, Schuller B. Deep Neural Networks for Acoustic Emotion Recognition: Raising the Benchmarks. in Proc of ICASSP. 2011; p. 5688–5691.

[13] Metallinou A, Lee S, Narayanan S. Decision Level Combination of Multiple Modalities for Recognition and Analysis of Emotional Expression. in Proc of ICASSP. 2010; p. 2462–2465.

[14] Polzehl T, Schmitt A, Metze F. Approaching multi-lingual emotion recognition from speech-on language dependency of acoustic prosodic features for anger detection. in Proc of Speech Prosody. 2010;.

[15] Bhaykar M, Yadav J, Rao KS. Speaker dependent, speaker independent and cross language emotion recognition from speech using GMM and HMM. in Communications (NCC), 2013 National Conference on IEEE. 2013; p. 1–5.

[16] Eyben F, Batliner A, Schuller B, Seppi D, Steidl S. Crosscorpus classification of realistic emotions—some pilot experiments. in Proc of the Third International Workshop on EMOTION (satellite of LREC). 2010;.

[17] Shami M, Verhelst W. Automatic classification of expressiveness in speech: A multi-corpus study. Speaker Classification II. 2007; p. 43–56. 10.1007/978-3-540-74122-0_5

[18] Neiberg D, Laukka P, Elfenbein HA. Intra-, inter-, and cross-cultural classification of vocal affect. in Proc of Speech Prosody. 2011;.

[19] Schuller B, Vlasenko B, Eyben F, Wllmer M, Stuhlsatz A, Wendemuth A, et al Cross-corpus acoustic emotion recognition: Variances and strategies. IEEE Transactions on Affective Computing. 2010;1(2):119–130. 10.1109/T-AFFC.2010.8

[20] Krizhevsky A, Sutskever I, Hinton GE. ImageNet Classification with Deep Convolutional Neural Networks. In: Pereira F, Burges CJC, Bottou L, Weinberger KQ, editors. Advances in Neural Information Processing Systems 25. Curran Associates, Inc.; 2012. p. 1097–1105.

[21] Abdel-Hamid O, Mohamed Ar, Jiang H, Deng L, Penn G, Yu D. Convolutional Neural Networks for Speech Recognition. IEEE/ACM Transactions on Audio, Speech, and Language Processing. 2014;22:1533–1545. 10.1109/TASLP.2014.2339736

[22] Sahidullah M, Saha G. Design, Analysis and Experimental Evaluation of Block Based Transformation in MFCC Computation for Speaker Recognition. Speech Communication. 2012;54 (4):543–565. 10.1016/j.specom.2011.11.004

[23] Bielefeld B. Language identification using shifted delta cepstrum. In Fourteenth Annual Speech Research Symposium. 1994;.

[24] Carrasquillo PAT, Singer E, Kohler MA, Greene RJ, Reynolds DA, Deller JR. Approaches to Language Identification using Gaussian Mixture Models and Shifted Delta Cepstral Features. in Proc of ICSLP2002-INTERSPEECH2002. 2002; p. 16–20.

[25] Sagha H, Matejka P, Gavryukova M, Povolný F, Marchi E, Schuller BW. Enhancing Multilingual Recognition of Emotion in Speech by Language Identification. in Proc of Interspeech. 2016; p. 2949–2953. 10.21437/Interspeech.2016-333

[26] Ranjan S, Yu C, Zhang C, Kelly F, Hansen JHL. Language recognition using deep neural networks with very limited training data. in Proc of ICASSP. 2016; p. 5830–5834.

[27] Lu X, Shen P, Tsao Y, Kawai H. Pair-wise Distance Metric Learning of Neural Network Model for Spoken Language Identification. in Proc of Interspeech. 2016; p. 3216–3220. 10.21437/Interspeech.2016-722

[28] Steidl S. Automatic Classification of Emotion-Related User States in Spontaneous Children’s Speech. Logos Verlag, Berlin. 2009;.

[29] Busso C, Bulut M, Lee CC, Kazemzadeh A, Mower E, Kim S, et al IEMOCAP: Interactive emotional dyadic motion capture database. Journal of Language Resources and Evaluation. 2008; p. 335–359. 10.1007/s10579-008-9076-6

[30] Burkhardt F, Paeschke A, Rolfes M, Sendlmeier W, Weiss B. A Database of German Emotional Speech. in Proc of Interspeech. 2005;.

[31] Heracleous P, Ishikawa A, Yasuda K, Kawashima H, Sugaya F, Hashimoto M. Machine Learning Approaches for Speech Emotion Recognition: Classic and Novel Advances. Computational Linguistics and Intelligent Text Processing—18th International Conference, CICLing 2017, Revised Selected Papers, Part II. 2017; p. 180–191.

[32] Li H, Ma B, Lee KA. Spoken language recognition: From fundamentals to practice. in Proc of the IEEE. 2013;101, no. 5:1136–1159. 10.1109/JPROC.2012.2237151

[33] Zissman MA. Comparison of Four Approaches to Automatic Language Identification of Telephone Speech. lEEE Transactions on Speech and Audio Processing. 1996;4(1):31–44. 10.1109/TSA.1996.481450

[34] Caseiro D, Trancoso I. Spoken Language Identification Using The Speechdat Corpus. In Proc of ICSLP’98. 1998;.

[35] Siniscalchi SM, Reed J, Svendsen T, Lee CH. Universal attribute characterization of spoken languages for automatic spoken language recognition. Computer speech and language. 2013;27:209–227. 10.1016/j.csl.2012.05.001

[36] Lee CH. Principles of Spoken Language Recognition. in Springer Handbook on Speech Processing and Speech Communication, J Benesty, Y Hunag M M Sondhi, Editors, SpringerVerlag. 2008;.

[37] Reynolds DA, Campbell WM, Shen W, Singer E. Automatic Language Recognition Via Spectral and Token Based Approaches. in Springer Handbook on Speech Processing and Speech Communication, J Benesty, Y Hunag M M Sondhi, Editors, SpringerVerlag. 2008;.

[38] Cole R, Inouye J, Muthusamy Y, Gopalakrishnan M. Language identification with neural networks: a feasibility study. in Proc of IEEE Pacific Rim Conference. 1989; p. 525–529.

[39] Leena M, Rao KS, Yegnanarayana B. Neural network classifiers for language identification using phonotactic and prosodic features. in Proc of Intelligent Sensing and Information Processing. 2005; p. 404–408.

[40] Montavon G. Deep learning for spoken language identification. in NIPS workshop on Deep Learning for Speech Recognition and Related Applications. 2009;.

[41] Moreno IL, Dominguez JG, Plchot O, Martinez D, Rodriguez JG, Moreno P. Automatic Language Identification Using Deep Neural Networks. in Proc of ICASSP. 2014; p. 5337–5341.

[42] Heracleous P, Takai K, Yasuda K, Mohammad Y, Yoneyama A. Comparative Study on Spoken Language Identification Based on Deep Learning. in Proc of EUSIPCO. 2018;.

[43] Jiang B, Song Y, Wei S, Liu JH, McLoughlin IV, Dai LR. Deep Bottleneck Features for Spoken Language Identification. PLos ONE. 2010;9(7):1–11. 10.1371/journal.pone.0100795 PMC4077656 24983963

[44] Zazo R, Diez AL, Dominguez JG, Toledano DT, Rodriguez JG. Language Identification in Short Utterances Using Long Short-Term Memory (LSTM) Recurrent Neural Networks. PLos ONE. 2016;11(1): e0146917 10.1371/journal.pone.0146917 26824467 PMC4732772

[45] Heracleous P, Mohammad Y, Takai K, Yasuda K, Yoneyama A. Spoken Language Identification Based on I-vectors and Conditional Random Fields. in Proc of IWCMC. 2018; p. 1443–1447.

[46] Reiter S, Schuller B, Rigoll G. Hidden Conditional Random Fields for Meeting Segmentation. in Proc of ICME. 2007; p. 639–642.

[47] Gunawardana A, Mahajan M, Acero A, Platt JC. Hidden Conditional Random Fields for Phone Classification. in Proc of Interspeech. 2005; p. 1117–1120.

[48] Llorens H, Saquete E, Colorado BN. TimeML Events Recognition and Classification: Learning CRF Models with Semantic Roles. in Proc of the 23rd International Conference on Computational Linguistics (Coling 2010). 2010; p. 725–733.

[49] Yu D, Wang S, Karam Z, Deng L. Language Recognition Using Deep-structured Conditional Random Fields. in Proc of ICASSP. 2010; p. 5030–5033.

[50] Cristianini N, Taylor JS. Support Vector Machines. Cambridge University Press, Cambridge 2000;.

[51] Dehak N, Carrasquillo PAT, Reynolds D, Dehak R. Language Recognition via Ivectors and Dimensionality Reduction. in Proc of Interspeech. 2011; p. 857–860.

[52] Shen P, Lu X, Liu L, Kawai H. Local Fisher Discriminant Analysis for Spoken Language Identification. in Proc of ICASSP. 2016; p. 5825–5829.

[53] Kim Y. Convolutional Neural Networks for Sentence Classification. in Proc of the 2014 Conference on Empirical Methods in Natural Language Processing (EMNLP). 2014; p. 1746–1751. 10.18653/v1/d16-1076 PMC5300751 28191551

[54] Rawat W, Wang Z. Deep Convolutional Neural Networks for Image Classification: A Comprehensive Review. Neural Communication. 2017;29:2352–2449. 10.1162/neco_a_00990 28599112

[55] Huynh XP, Tran TD, Kim YG. Convolutional Neural Network Models for Facial Expression Recognition Using BU-3DFE Database In: Kim K, Joukov N, editors. Information Science and Applications (ICISA) 2016. Lecture Notes in Electrical Engineering. vol. 376 Springer; 2013 p. 441–450. 10.1007/978-981-10-0557-2_44

[56] Lim W, Jang D, Lee T. Speech Emotion Recognition Using Convolutional and Recurrent Neural Networks. in Proc of Signal and Information Processing Association Annual Summit and Conference (APSIPA). 2016.

[57] Ganapathy S, Han K, Thomas S, Omar M, Segbroeck MV, Narayanan SS. Robust Language Identification Using Convolutional Neural Network Features. in Proc of Interspeech. 2014;.

[58] Hansen JHL, Bořil H. On the issues of intra-speaker variability and realism in speech, speaker, and language recognition tasks. Speech Communication. 2018;101:94–108. 10.1016/j.specom.2018.05.004

[59] Lee CC, Mower E, Busso C, Lee S, Narayanan S. Emotion recognition using a hierarchical binary decision tree approach. Speech Communication. 2011;53:1162–1171. 10.1016/j.specom.2011.06.004

[60] Lee J, Tashev I. High-level Feature Representation using Recurrent Neural Network for Speech Emotion Recognition. in Proc of Interspeech. 2015; p. 1537–1540.

[61] Lakomkin E, Weber C, Magg S, Wermter S. Reusing Neural Speech Representations for Auditory Emotion Recognition. in Proc the 8th International Joint Conference on Natural Language Processing. 2017; p. 423–430.

[62] Shen L, Wang W. Improving Speech Emotion Recognition Based on ToBI Phonological Representations. in PATTERNS 2018: The Tenth International Conference on Pervasive Patterns and Applications. 2018; p. 1–5.

[63] Attabi Y, Alam J, Dumouchel P, Kenny P, Shaughnessy DO. Multiple Windowed Spectral Features for Emotion Recognition. in Proc of ICASSP. 2013; p. 7527–7531.

[64] Cao H, Verma R, Nenkova A. Combining Ranking and Classification to Improve Emotion Recognition in Spontaneous Speech. in Proc of INTERSPEECH. 2012;.

[65] Le D, Provost EM. Emotion Recognition From Spontaneous Speech Using Hidden Markov Models With Deep Belief Networks. in Proc of IEEE ASRU. 2013; p. 216–221.

[66] Cabaleiro EP, Costantini G, Batliner A, Baird A, Schuller B. Categorical vs Dimensional Perception of Italian Emotional Speech. in Proc of Interspeech. 2018; p. 3638–3642. 10.21437/Interspeech.2018-47

